# Clinical effects of a 0.5% selenium sulfide shampoo on dandruff and scalp microbiota in adolescents: a prospective single-arm clinical study

**DOI:** 10.3389/fmed.2026.1866777

**Published:** 2026-07-23

**Authors:** Wencai Jiang, Rui Zhang, Liwei Wang, Yafei Xu, Yimei Tan

**Affiliations:** 1 Department of Skin and Cosmetic Research, Shanghai Skin Disease Hospital, School of Medicine, Tongji University, Shanghai, China; 2 Professional Technical Service Platform for Clinical Evaluation of Skin Health Related Products, Shanghai Science and Technology Commission (21DZ2294500), Shanghai, China; 3 School of Life Science, Fudan University, Shanghai, China

**Keywords:** adolescents, dandruff, scalp microbiome, seborrheic dermatitis, selenium sulfide

## Abstract

**Introduction:**

This prospective, exploratory single-arm pilot study evaluated the clinical efficacy, safety, and scalp microecological effects of a 0.5% selenium sulfide shampoo in adolescents with dandruff.

**Methods:**

Following a 2-week washout, 31 adolescents applied the shampoo three to four times weekly for 4 weeks. Investigator assessments, patient-reported pruritus scores, instrumental assessments (transepidermal water loss, stratum corneum hydration, sebum spot size), and scalp microbiome analysis were performed at baseline (week 0) and at week 4 (or week 2 for some measures).

**Results:**

Investigator assessments showed significant improvements: median dandruff scores declined from 16 to 6, erythema scores from 12 to 2, total scalp oiliness scores from 153 to 127, and total hair lift scores increased from 107 to 122 at week 4. Patient-reported pruritus scores decreased from 109 at baseline to 60, 48, and 37 at weeks 1, 2, and 4, respectively (P<0.001). Instrumental assessments demonstrated reduced transepidermal water loss (35.66 ± 8.18 to 31.12 ± 4.36 g/m^2^·h, *P* < 0.01), increased stratum corneum hydration (30.49 ± 10.28 to 35.01 ± 9.64 a.u., *P* < 0.001), and decreased sebum spot size at week 2 (953.65 ± 892.50 to 692.33 ± 456.19 pixels, *P* < 0.05). Genus-level microbiome profiling revealed significantly elevated *Cutibacterium* abundance (*P* < 0.001), alongside reduced levels of *Staphylococcus* and *Malassezia* (both *P* < 0.01). No adverse events were reported.

**Conclusion:**

The 0.5% selenium sulfide shampoo significantly improved clinical symptoms of dandruff in adolescents, with favorable safety, barrier-protective effects, promoted beneficial compositional shifts in the scalp microbial community toward a healthier profile.

## 1 Introduction

Pityriasis capitis, commonly referred to as dandruff, is a common scalp disorder characterized by excessive desquamation of the scalp and is widely regarded as a mild clinical manifestation of seborrheic dermatitis (1, 2). Although not life-threatening, the condition can substantially impair quality of life owing to persistent scaling, visible flaking, and associated discomfort (2). Dandruff affects individuals across all age groups, including infants, adolescents, and adults, and is estimated to affect approximately 50% of the global population, with a higher prevalence reported in males than in females (3). Despite its widespread occurrence, epidemiologic data focusing specifically on adolescents remain limited. Nevertheless, available studies from France and Nigeria have consistently demonstrated that adolescents represent the age group with the highest prevalence of dandruff (4, 5). Seborrheic dermatitis itself has an estimated global prevalence of approximately 4.38% (6). Population-based studies further support the high burden of scalp seborrheic conditions among adolescents: a prevalence of 11% has been reported among adolescent males in Pelotas, Brazil (7), while a similar prevalence of 10.17% has been documented among Han Chinese adolescents aged 12–20 years (8). Collectively, these findings highlight dandruff as a particularly prevalent dermatologic concern during adolescence and underscore the need for effective and well-tolerated management strategies in this population.

Beyond its physical manifestations, dandruff and related scalp disorders can exert a substantial psychosocial burden, particularly among adolescents. The visible nature of scalp flaking and desquamation, often adhering to hair shafts and dark clothing, can lead to embarrassment, diminished self-confidence, and social discomfort in this age group (9, 10). Despite its high prevalence and adverse impact on psychosocial health, the pathogenesis of dandruff and seborrheic dermatitis remains incompletely understood. Current evidence suggests that the condition arises from a multifactorial interaction involving sebaceous gland activity, colonization by *Malassezia* yeasts, and individual host susceptibility, including variations in skin barrier function and immune response (11). Specifically in adolescents, puberty-related hormonal changes stimulate excessive sebaceous gland secretion, which significantly predisposes this population to scalp seborrheic conditions. Among these factors, members of the genus *Malassezia* are widely considered central to disease development and exacerbation, as their metabolic activity and interactions with host skin lipids can trigger inflammatory responses and abnormal keratinocyte turnover, ultimately leading to the characteristic scaling observed in dandruff.

However, current therapeutic options for dandruff present several limitations, particularly with respect to formulation concentration. Selenium sulfide-containing shampoos are widely used over-the-counter antifungal treatments for dandruff and seborrheic dermatitis, and have been explicitly recommended for adolescent patients by recent clinical consensus guidelines (12). However, commercially available formulations typically contain concentrations ranging from 1% to 2.5%. Although these formulations are effective, higher concentrations have been associated with undesirable adverse effects, including scalp dryness, hair shaft dryness, textural alterations, and chemical irritation (13, 14). These concerns are particularly relevant among adolescents, as their scalp skin barrier may already be compromised in the presence of dandruff, and this vulnerability may be further accentuated during puberty (15). Consequently, the use of high-concentration antifungal shampoos may negatively affect treatment adherence owing to unfavorable cosmetic outcomes, such as excessive dryness and dull hair appearance. Despite these considerations, evidence regarding the efficacy and tolerability of lower-concentration selenium sulfide formulations specifically designed for adolescents remains limited.

Accordingly, the present study aimed to evaluate the clinical efficacy and safety of a 0.5% selenium sulfide shampoo in adolescents with dandruff. In addition to assessing improvements in clinical symptoms, this study sought to determine whether a lower-concentration formulation could effectively control dandruff and scalp sebum while maintaining a favorable safety profile and improved cosmetic acceptability, thereby potentially enhancing treatment adherence in this sensitive population.

## 2 Materials and methods

### 2.1 Participants

This prospective, exploratory, single-arm pilot study was conducted at Shanghai Skin Diseases Hospital, Shanghai, China, between July 6, 2025, and August 17, 2025 to evaluate adolescents presenting with dandruff. A self-controlled design was adopted due to ethical considerations regarding the prolonged withholding of symptomatic relief in minors, as well as the adherence risks associated with placebo assignment in this population. Eligible participants were males or females aged 12–17 years with clinically evident dandruff, defined as an Adherent Scalp Flaking Scale (ASFS) score ≥12, including individuals with mild-to-moderate seborrheic dermatitis, and a scalp erythema score between 1 and 6. Participants were required to have abstained from hair dyeing, perming, styling, or other chemical hair treatments for at least 1 month prior to enrollment, and to be free of other scalp and hair conditions that affect study outcomes. The study protocol was approved by the Ethics Committee of Shanghai Skin Diseases Hospital (approval number: 2025-23-C) and conducted in accordance with the Declaration of Helsinki. Written informed consent was obtained from the legal guardians, and written assent was obtained from all adolescent participants prior to enrollment.

### 2.2 Procedure

The study protocol comprised a 2-week washout period followed by a 4-week treatment phase. During the washout period, participants were instructed to discontinue all prior medicated or anti-dandruff hair products and instead use a neutral shampoo provided by the study team three to four times per week to minimize potential confounding effects of previous treatments. Participants who continued to meet the eligibility criteria (specifically, maintaining a minimum ASFS score of 12) at the end of the washout period proceeded to the treatment phase, during which they applied the investigational shampoo at a frequency of three to four times weekly for four consecutive weeks. The investigational product was formulated at a physiologically compatible weakly acidic pH of 5.1. Its vehicle comprised a surfactant system (sodium C14-16 olefin sulfonate, sodium lauroyl sarcosinate, lauryl hydroxysultaine, sodium lauroamphoacetate, and cocamidopropyl betaine), preservatives (sodium benzoate and phenoxyethanol), and fragrance (parfum). The comprehensive ingredient list is detailed in Supplementary Table S6. Clinical and instrumental assessments were conducted at four predefined visits: baseline (week 0) and follow-up visits at weeks 1, 2, and 4. To standardize scalp conditions prior to evaluation, participants were required to wash their hair once within 48 ± 2 h before each study visit, to refrain from using any other hair care products, and to refrain from combing their hair on the day of assessment. Upon arrival at the study site, participants loosened their hair and underwent a 30-min acclimatization period in a controlled laboratory environment with standardized temperature (18–22 °C) and related humidity (40%−60%) before undergoing investigator-assessed clinical evaluations, noninvasive instrumental measurements, and subject-reported outcome assessments.

### 2.3 Assessments

**Figure 1:**
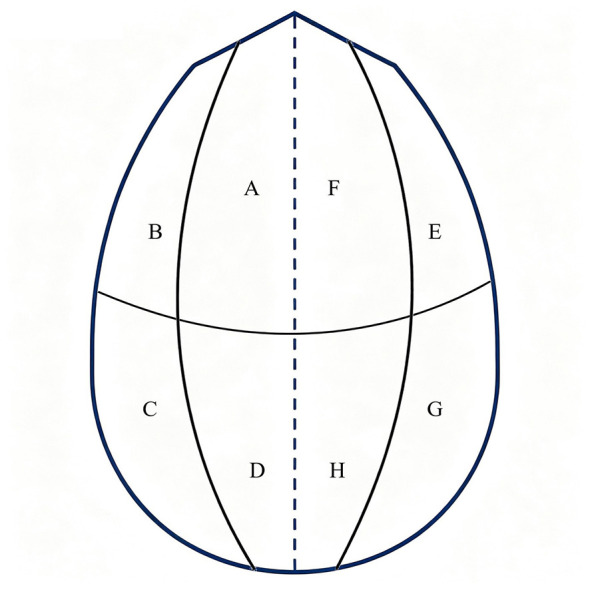
Division of scalp regions for clinical evaluation. Schematic illustration of the eight predefined scalp regions used for clinical assessment of dandruff severity and scalp erythema [Adapted from reference (16)].

Clinical evaluations were performed at week 0 and at weeks 1, 2, and 4 by trained investigators using standardized scoring systems under standardized lighting conditions. Dandruff severity was assessed using the ASFS (0–10 points per region, yielding a maximum total score of 80) (16). Scalp erythema, scalp oiliness, and hair lift were evaluated using internally developed 0–9 ordinal scales, where lower scores indicated improvement for erythema and oiliness, and higher scores reflected improved hair lift. Prior to the formal trial, all assessors received unified training, and acceptable intra-rater and inter-rater reliability for these in-house scales was verified. The hair lift score was evaluated using an internally developed 10-point visual grading scale assessing hair volume and inter-fiber space (17). The detailed scoring criteria and anchor descriptions for all clinical parameters are provided in Supplementary Table S7. For dandruff and erythema assessments, the scalp was divided into eight predefined anatomical regions, each scored independently (Figure 1); cumulative scores were subsequently used for statistical analyses. Safety evaluations were conducted at each visit using a four-point grading scale (0–3) to document treatment-related adverse events, including edema, dryness, stinging, pruritus, and papules (0 = none; 1 = mild; 2 = moderate; 3 = severe). Patient-reported outcomes were also collected. Participants rated the intensity of scalp pruritus using a Numeric Rating Scale (NRS) immediately after product use (2 h) and at weeks 1, 2, and 4 (0 = no itch, 10 = worst imaginable itch). Perceived treatment benefits and sensory attributes of the product were assessed using a five-point Likert scale ranging from strongly disagree (1) to strongly agree (5).

In addition to clinical assessments, instrument-based measurements were performed at each study visit to objectively characterize scalp condition. High-resolution images of the two scalp regions exhibiting the most severe flaking were captured using a Skin-Visioscan^®^ VC20Plus imaging system. Scalp sebum levels were quantified using Sebufix^®^ F16 adhesive tapes, with analysis of the percentage sebum spot area and average sebum spot size. Surface characteristics of the most erythematous scalp area were documented using dermatoscopy (I-scope, × 30 magnification). Transepidermal water loss (TEWL) was measured using a Tewameter^®^ TM Nano, and stratum corneum hydration was assessed using a Corneometer (Pin Probe, Cortex Technology).

### 2.4 Scalp microbiota analysis

Scalp microbiome samples were collected from predefined lesional scalp areas using a standardized swab-based sampling technique. Samples were obtained at baseline prior to treatment initiation and again after 4 weeks of treatment. Each sampling area (approximately 5 × 0.6 cm^2^) was swabbed back and forth for 20 passes, and three swabs were collected from each site with approximately 1 cm spacing between collection points. Following collection, swab heads were aseptically transferred into sterile centrifuge tubes and stored at −80 °C until further processing.

Microbial DNA was extracted from samples using an HiPure Stool/Soil DNA Mini Kit (Magen), according to the manufacturer's protocols. The Qubit 4 (Thermo Fisher, United States) was used to determine the final DNA concentration and purity, and DNA quality was checked using 1% agarose gel electrophoresis. For bacteria, the V3–V4 hypervariable regions of the 16S rRNA gene were amplified using the primers 338F (5′-ACTCCTACGGGAGGCAGCAG-3′) and 806R (5′-GGACTACHVGGGTWTCTAAT-3′) with a GeneAmp 9700 thermocycler PCR system (ABI, Foster City, CA, United States). We targeted the V3–V4 hypervariable region for broad bacterial profiling, as this segment is standard for human microbiome Illumina paired-end amplicon sequencing and well-annotated in curated databases including SILVA. For fungi, the internal transcribed spacer (ITS) region was amplified using the primer pair ITS1F (5′-CTTGGTCATTTAGAGGAAGTAA-3′) and ITS2R (5′-GCTGCGTTCTTCATCGATGC-3′).

The PCR product was extracted by 2% agarose gel and purified using an AxyPrep DNA Gel Extraction Kit (Axygen Biosciences, Union City, CA), according to the manufacturer's instructions, and quantified using Qubit 4 (Thermo Fisher, United States). Purified amplicons were pooled in equimolar and paired-end sequenced on the Illumina Novaseq 6000 PE250 platform (Illumina, San Diego, United States) according to the standard protocols by Honsunbio Technology Co. Ltd (Shanghai, China).

Raw paired-end 16S rRNA V3–V4 amplicon reads were processed via DADA2 within QIIME 2 to generate amplicon sequence variants (ASVs), with taxonomic classification performed against the SILVA 138.2 classifier optimized for the 338F/806R primer pair. Given the limited species-level resolution of this hypervariable region for closely related skin bacteria-especially Staphylococcus-all 16S datasets were analyzed at the genus rank. Raw ITS amplicon reads underwent identical QIIME 2/DADA2 workflows for ASV inference, followed by taxonomic annotation using the UNITE fungal classifier (release 2025-02-19).

### 2.5 Statistical analysis

Statistical analyses were conducted using SPSS Statistics version 22.0 (IBM Corp., Armonk, NY, USA) and R version 4.5.1. Continuous variables were summarized as mean ± standard deviation. Data distribution was assessed using the Shapiro-Wilk test for normality. For normally distributed variables, changes between baseline and post-treatment measurements were analyzed using paired Student's *t*-tests. When the assumption of normality was not met, comparisons were performed using the Wilcoxon signed-rank test. Ordinal variables were analyzed using the Wilcoxon signed-rank test. Responses from self-assessment questionnaires were evaluated using a binomial test with a reference proportion of 0.50. All statistical tests were two-sided, and a *P* value of less than 0.05 was considered to indicate statistical significance.

## 3 Results

### 3.1 Demographic and baseline characteristics

A total of 42 adolescents were initially enrolled during the washout phase of the study. Of these, six participants did not meet the eligibility criteria at the start of the treatment phase (due to sub-threshold dandruff severity with an ASFS score < 12 at the baseline evaluation) and were therefore excluded. One participant withdrew during follow-up for personal reasons. Consequently, 35 participants completed the intervention period. Four additional participants were excluded from the final analysis because of non-compliance with the study protocol, resulting in a final analysis cohort of 31 participants. The study population comprised 15 males (51.6%) and 16 females (48.4%), with ages ranging from 13 to 17 years and a median age of 15 years. Baseline clinical characteristics of the study cohort are summarized in Table 1.

**Table 1:** Baseline characteristics of the study participants.

Characteristic	Total (*N* = 31)
Age, years, median (range)	15 (13-17)
Sex, *n* (%)	
Female	15 (48.4)
Male	16 (51.6)
Dandruff severity (ASFS score), median (range)	16 (12-40)
Scalp erythema score, median (range)	12 (6-26)

ASFS, adherent scalp flaking scale.

### 3.2 Clinical efficacy

**Figure 2:**
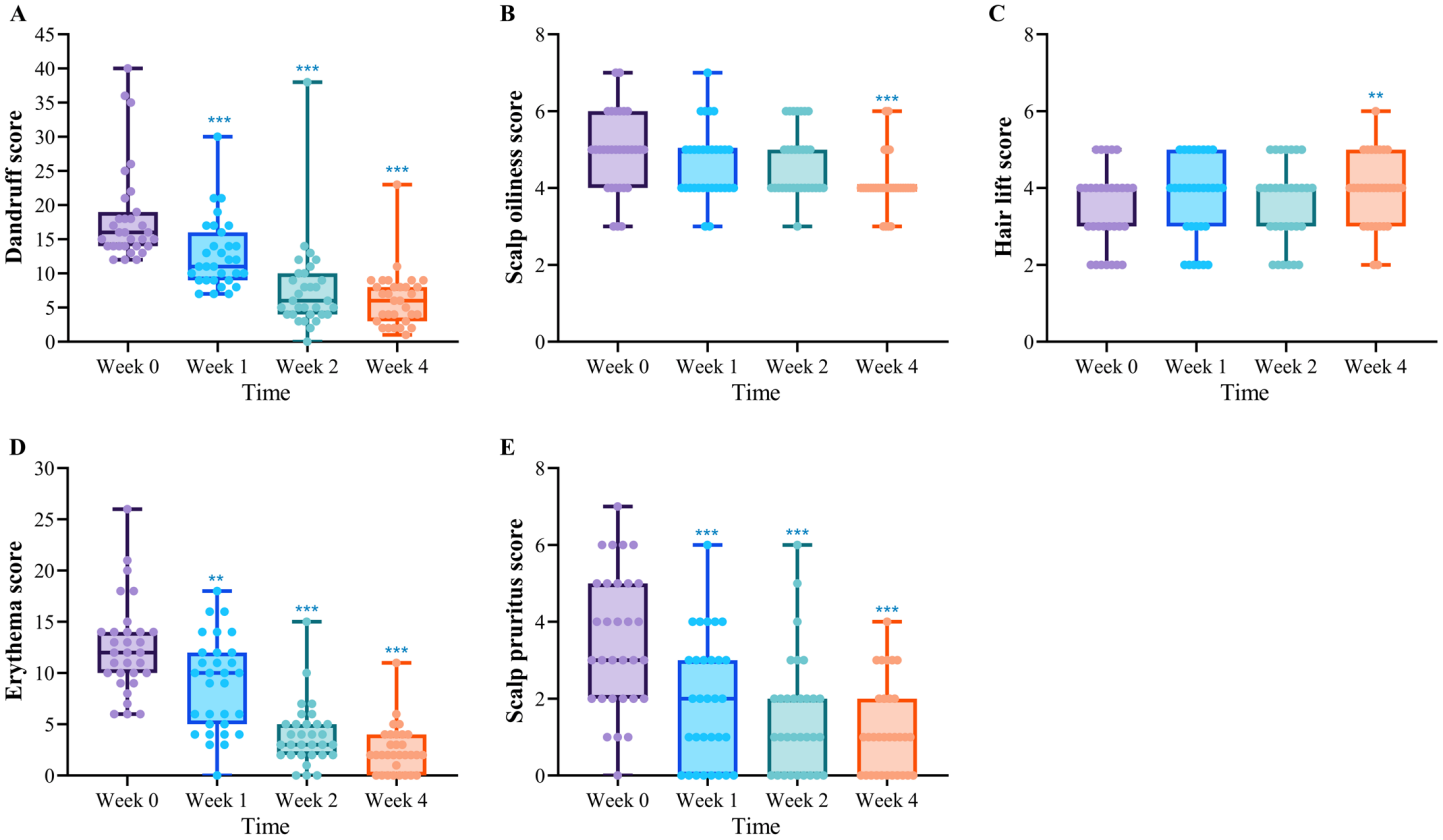
Changes in clinical efficacy parameters during the 4-week treatment period. **(A)** Dandruff score. **(B)** Scalp oiliness score. **(C)** Hair lift score. **(D)** Erythema score. **(E)** Scalp pruritus score. ** *P* < 0.01; *** *P* < 0.001 compared with baseline.

Investigator assessments demonstrated significant improvements across all evaluated clinical parameters over the 4-week treatment period (Figure 2, Supplementary Table S1). Reductions in dandruff severity and scalp erythema were evident as early as week 1 and continued throughout the study. The total dandruff score decreased from 566 at baseline to 194 at week 4, with the median score declining from 16 to 6. Similarly, the total erythema score decreased markedly from 387 at baseline to 77 at week 4, with the median score declining from 12 to 2, reflecting substantial attenuation of scalp inflammation.

Improvements in scalp oiliness and hair lift became statistically significant at week 4. The total scalp oiliness score decreased from 153 at baseline to 127 at week 4, indicating improved oil control, whereas the hair lift score increased from 107 to 122 over the same period, suggesting enhanced hair volume and scalp cleanliness.

Patient-reported outcomes were consistent with investigator assessments. The total pruritus score measured using the NRS decreased from 109 at baseline to 60, 48, and 37 at weeks 1, 2, and 4, respectively, representing a significant reduction in itch intensity (*P* < 0.001). Self-assessment questionnaires further indicated high levels of perceived benefit and product acceptability (Supplementary Table S2). A large majority of participants reported improvements in dandruff, itch relief, scalp comfort, and oil control, with recognition rates for several key efficacy indicators exceeding 90%. Additionally, overall satisfaction with product performance, including perceived mildness and ease of rinsing, remained consistently above 90% throughout the study period.

### 3.3 Instrument-based measurements

**Figure 3:**
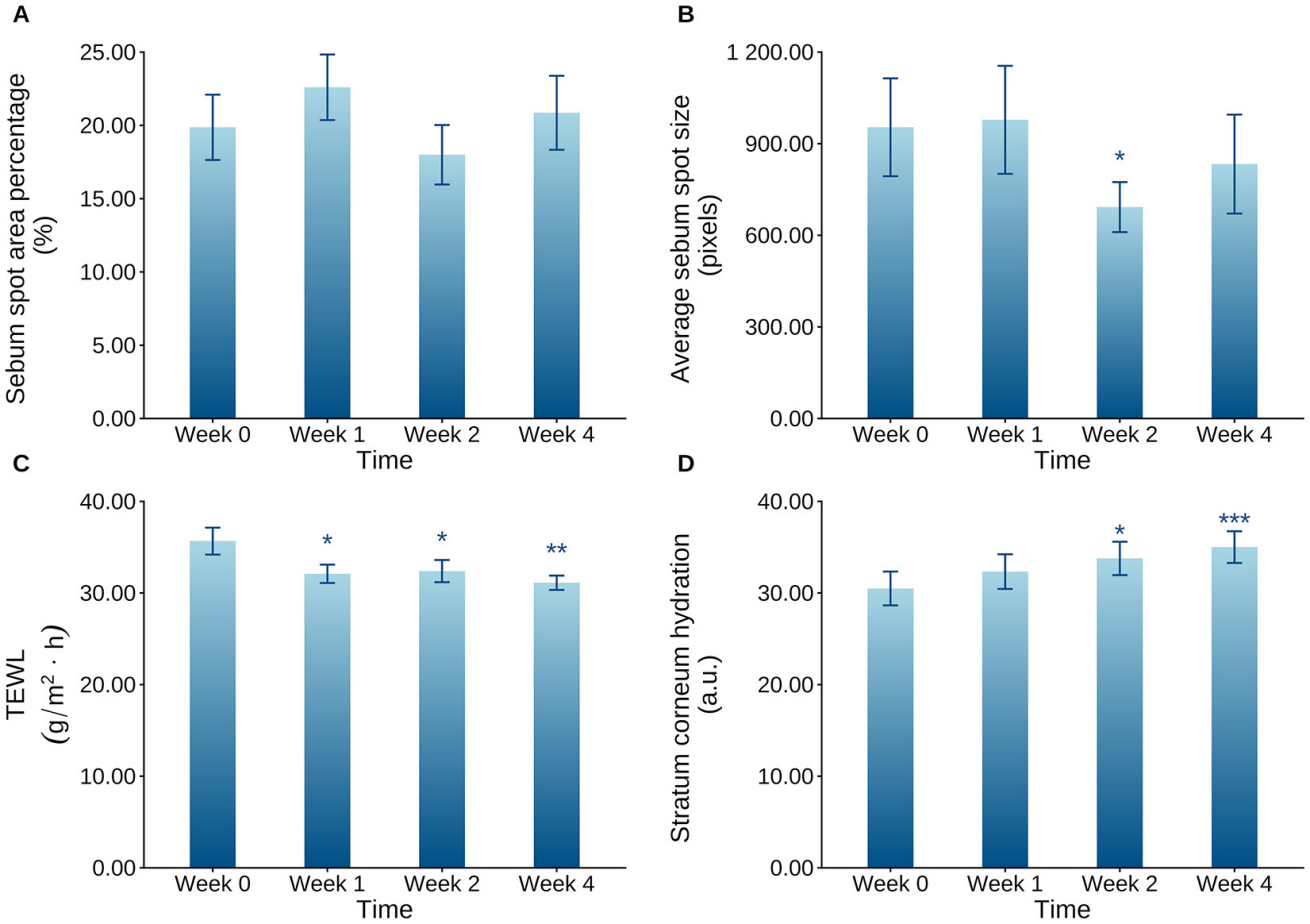
Changes in instrument-based scalp measurements during the 4-week treatment period. **(A)** Sebum spot area percentage (%). **(B)** Average sebum spot size (pixels). **(C)** Transepidermal water loss (g/m^2^·h). **(D)** Stratum corneum hydration (a.u.). * *P* < 0.05; ** *P* < 0.01; *** *P* < 0.001 compared with baseline.

Instrument-based assessments further supported the clinical improvements observed during the treatment period (Figure 3, Supplementary Table S3). Sebufix analysis indicated changes in the distribution pattern of scalp sebum; however, no statistically significant reduction in the total sebum spot area was detected over the 4-week period. Notably, the average sebum spot size decreased significantly at week 2, declining from 953.65 ± 892.50 pixels at baseline to 692.33 ± 456.19 pixels (*P* < 0.05), suggesting a reduction in localized sebum accumulation.

Measures of scalp barrier function demonstrated consistent improvement throughout the study. TEWL decreased significantly at all follow-up time points, declining from 35.66 ± 8.18 g/m^2^·h at baseline to 31.12 ± 4.36 g/m^2^·h at week 4 (*P* < 0.01). Concurrently, stratum corneum hydration increased progressively, rising from 30.49 ± 10.28 a.u. at baseline to 33.77 ± 10.14 a.u. at week 2 (*P* < 0.05) and further to 35.01 ± 9.64 a.u. at week 4 (*P* < 0.001). Representative imaging findings, including skin scanner images of dandruff-affected scalp areas and dermoscopic images of erythematous regions, are presented in Supplementary Figures S1, S2, respectively.

### 3.4 Scalp microbiota analysis

**Figure 4:**
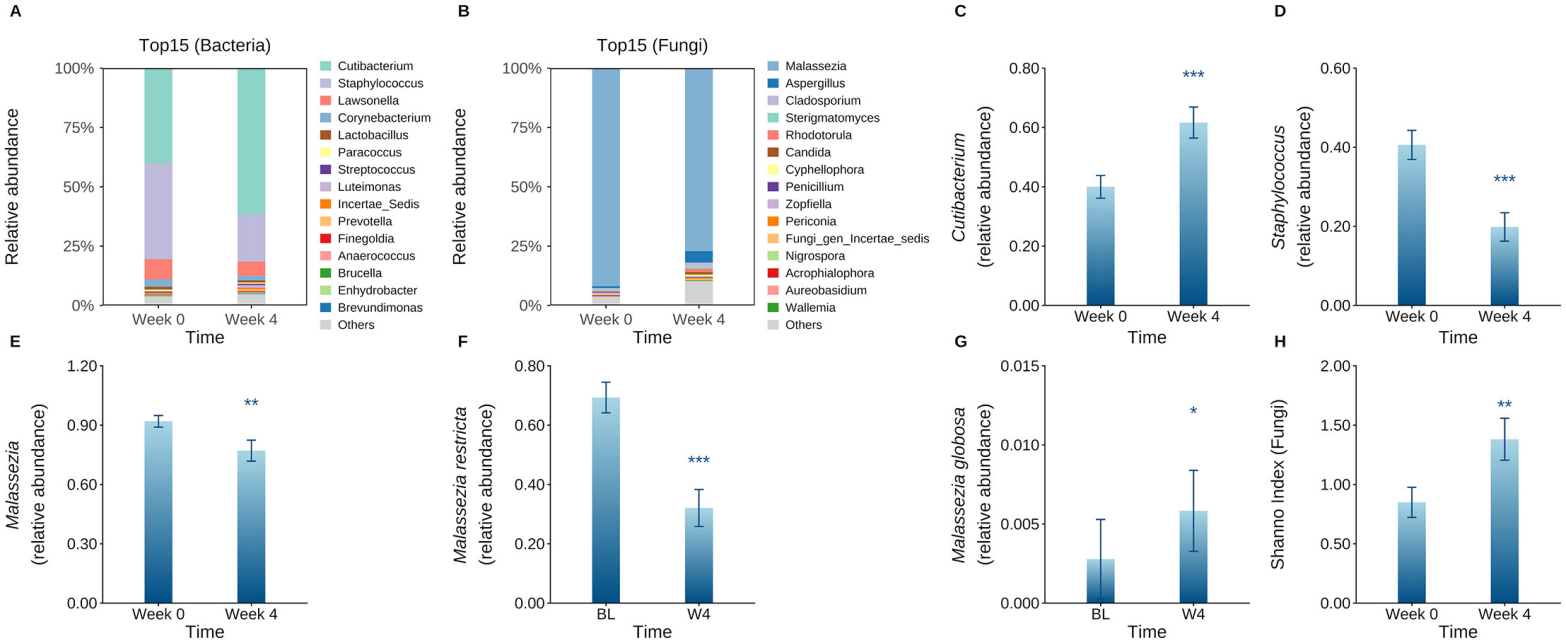
Changes in the relative abundance and diversity of dominant scalp microbial taxa following treatment at baseline (BL) and week 4 (W4). **(A)** Stacked bar chart showing the relative abundance of the top 15 bacterial taxa. **(B)** Stacked bar chart showing the relative abundance of the top 15 fungal taxa. **(C)** Relative abundance of *Cutibacterium*. **(D)** Relative abundance of *Staphylococcus*. **(E)** Relative abundance of the *Malassezia* genus. **(F)** Relative abundance of *Malassezia restricta*. **(G)** Relative abundance of *Malassezia globosa*. **(H)** Fungal alpha diversity (Shannon index). ** *P* < 0.01; *** *P* < 0.001 compared with baseline.

Analysis of the scalp microbiome revealed treatment-associated changes in both bacterial and fungal community composition following treatment. At baseline, the bacterial microbiota was dominated by *Cutibacterium* and *Staphylococcus*, with relative abundances of approximately 40% and 41%, respectively. After 4 weeks of product use, *Cutibacterium* increased significantly to 62%, whereas *Staphylococcus* decreased to 20% (both *P* < 0.001; Figures 4A, C, D and Supplementary Table S4). Bacterial alpha diversity also decreased after treatment. The Shannon index declined from 2.08 ± 1.17 at baseline to 1.81 ± 1.45 at week 4 (*P* < 0.01), the Simpson index decreased from 0.60 ± 0.17 to 0.46 ± 0.25 (*P* < 0.001), and the Pielou evenness index decreased from 0.38 ± 0.13 to 0.31 ± 0.18 (*P* < 0.01) (Supplementary Table S5). Beta diversity analysis also revealed significant differences in microbial community structure between baseline and week 4, as supported by Anosim (R = 0.152, *P* = 0.001), Multiple Response Permutation Procedure (MRPP) (A = 0.06, *P* = 0.001), and Adonis2 (R^2^ = 0.097, F = 7.237, *P* = 0.001).

Fungal community composition also changed after treatment, particularly within the genus *Malassezia* (Figure 4B). The total relative abundance of *Malassezia* decreased significantly (Figure 4E). At the species level, *Malassezia restricta* decreased markedly from 0.69 ± 0.31 at baseline to 0.32 ± 0.37 at week 4 (*P* < 0.001; Figure 4F), whereas *Malassezia globosa* increased from 0.003 ± 0.015 to 0.006 ± 0.015 (*P* < 0.05; Figure 4G and Supplementary Table S4). In contrast to the bacterial community, fungal alpha diversity increased after treatment. The Shannon index rose from 0.85 ± 0.75 at baseline to 1.38 ± 1.05 at week 4 (*P* < 0.01, Figure 4H), accompanied by increases in the Simpson index (0.35 ± 0.26 to 0.47 ± 0.28, *P* < 0.05) and the Pielou evenness index (0.23 ± 0.17 to 0.33 ± 0.21, *P* < 0.05) (Supplementary Table S5). Fungal beta diversity analysis showed significant differences between baseline and week 4 samples, with consistent results from ANOSIM (R = 0.218, *P* = 0.001), MRPP (A = 0.084, *P* = 0.001), and Adonis2 analysis (R^2^ = 0.136, F = 10.748, *P* = 0.001).

Additional ratio analyses were performed to compare these microbial shifts with scalp microbiome markers reported in previously studies. The genus-level *Cutibacterium/Staphylococcus* ratio increased significantly from 2.41 ± 4.61 at baseline to 38.21 ± 116.03 at week 4 (*P* < 0.001). Since *Malassezia globosa* showed zero relative abundance in several samples at the species level, a pseudocount of 0.001 was added to both taxa prior to log_2_ transformation to calculate the *Malassezia restricta/Malassezia globosa* ratio, defined as log_2_[(0.001 + *M. restricta*)/(0.001 + *M. globosa*)]. The ratio decreased significantly from 8.68 ± 1.82 to 5.76 ± 2.99 (*P* < 0.001) (Supplementary Table S8 and Supplementary Figure S3).

### 3.5 Safety

No cases of edema, dryness, or stinging were reported at any assessment time point (Table 2). Notably, scalp-related adverse symptoms improved during treatment. Scores for pruritus and papules demonstrated significant reductions as early as week 1 and continued to decline over the course of the study.

**Table 2:** Safety outcomes during the 4-week treatment period.

Parameter	Week 0	Week 1	Week 2	Week 4
Edema	0 (0, 0)	0 (0, 0)	0 (0, 0)	0 (0, 0)
Dryness	0 (0, 0)	0 (0, 0)	0 (0, 0)	0 (0, 0)
Stinging	0 (0, 0)	0 (0, 0)	0 (0, 0)	0 (0, 0)
Pruritus	1 (0, 2)	0 (0, 2)^***^	0 (0, 1)^***^	0 (0, 1)^***^
Papules	0 (0, 1)	0 (0, 1)^**^	0 (0, 1)^**^	0 (0, 0)^***^

Data are presented as median (minimum, maximum).

^**^P < 0.01; ^***^ *P* < 0.001, compared with baseline (week 0).

## 4 Discussion

The present study demonstrates that a 0.5% selenium sulfide shampoo provides meaningful clinical benefits for adolescents with dandruff, representing, to our knowledge, one of the first clinical evaluations of a low-concentration selenium sulfide formulation specifically in this population. Treatment was associated with marked improvements in key clinical manifestations of dandruff, including scaling, scalp oiliness, erythema, and pruritus, accompanied by high levels of user-reported satisfaction and tolerability. In addition to its established antifungal activity and ability to regulate corneocyte turnover, the formulation appeared to modulate the scalp microenvironment, as reflected by alterations in microbial community composition, including suppression of *Malassezia* species and shifts in bacterial taxa. Furthermore, instrumental measurements indicated improvements in scalp barrier function, evidenced by reductions in TEWL and increases in stratum corneum hydration. Collectively, these findings suggest that a 0.5% selenium sulfide shampoo may provide an effective and well-tolerated therapeutic option for managing dandruff and related seborrheic scalp conditions in adolescents, while also contributing to exploratory modulation of scalp microbial community structure and improvement of barrier integrity.

Selenium sulfide is a well-established antifungal agent widely recommended for the management of dandruff and seborrheic dermatitis, with multiple clinical studies supporting its therapeutic efficacy. Formulations containing 2.5% selenium sulfide have demonstrated substantial clinical benefit in patients with moderate-to-severe dandruff, and their safety and effectiveness have been confirmed in clinical cohorts (13, 18). Similarly, 1% selenium sulfide shampoos have been evaluated in prospective and real-world observational studies in adults, which consistently report favorable clinical outcomes together with good tolerability profiles (19–22). Building on these findings, the present study evaluated a lower-concentration formulation (0.5%) and demonstrated that meaningful therapeutic improvements can also be achieved in the previously understudied adolescent population. Specifically, significant reductions in dandruff severity, scalp erythema, and pruritus were observed, with clinical improvement in dandruff apparent as early as week 1, consistent with the rapid onset of action reported in previous studies of selenium sulfide-based therapies (18, 23). With regard to sebum regulation, our findings align with earlier reports showing no statistically significant reduction in overall sebum production after 2 or 4 weeks of treatment measured by sebumetry (18, 22). Although the total sebum spot area did not change significantly, a reduction in average sebum spot size was observed, suggesting a normalization trend in sebaceous gland activity. Notably, instrumental assessments and participant-reported outcomes further indicated that the low-concentration formulation was associated with reduced TEWL and increased stratum corneum hydration. These findings suggest that a 0.5% selenium sulfide shampoo may provide a milder therapeutic approach that preserves scalp barrier integrity while maintaining clinically meaningful efficacy.

Alterations in the scalp microbiota may represent an important mechanism underlying the therapeutic effects and barrier improvements observed in this study. In healthy individuals, the scalp bacterial community is typically dominated by the genera *Cutibacterium* and *Staphylococcus* (24). Previous studies have reported that individuals with dandruff exhibit microbial dysbiosis characterized by increased abundance of *Staphylococcus* and decreased abundance of *Cutibacterium* (25, 26). In the present study, treatment with the 0.5% selenium sulfide shampoo significantly reversed this dysbiotic pattern, driving a marked increase in *Cutibacterium* and a concurrent decrease in *Staphylococcus* at the genus level. Although previous investigations have characterized health-associated bacterial signatures at the species level (e.g., *Staphylococcus epidermidis*) (25), precise species-level discrimination within cutaneous *Staphylococcus* remains a recognized limitation of short-read 16S (V3–V4) amplicon sequencing. As highlighted by methodological studies, skin microbiome profiles are inherently influenced by experimental design, particularly the choice of targeted hypervariable regions (27). Consequently, our bacterial findings are objectively interpreted at the genus level. In contrast, the internal transcribed spacer (ITS) sequencing methodology employed herein provides robust species-level resolution for the fungal community.

Building upon this species-level resolution, our analysis revealed substantial modulation within the fungal community, particularly among *Malassezia* species, which are widely recognized as key etiological agents in dandruff pathogenesis. A previous study conducted in Korea demonstrated that the abundance of *Malassezia restricta* is elevated in individuals with seborrheic dermatitis, whereas *Malassezia globosa* is comparatively reduced (28). Consistent with these findings, the present study demonstrated a marked reduction in *M. restricta* and a significant increase in *M. globosa* following treatment. Recent comprehensive studies confirm that clinical improvement in dandruff severity is robustly associated with this exact macro-ecological signature—the enrichment of *Cutibacterium* and the reduction of *Staphylococcus* at the genus level, alongside fungal shifts characterized by decreased *M. restricta* and increased *M. globosa* (29, 30). Furthermore, the observed microbial diversity patterns support this interpretation. Specifically, bacterial alpha-diversity and evenness decreased after treatment, whereas fungal alpha-diversity and evenness increased. Beta diversity analysis revealed significant separation between baseline and week 4 samples for both bacterial and fungal communities, indicating a global restructuring of the scalp microbiome following intervention. Notably, the beta diversity changes in the fungal community were more pronounced, with an effect size approximately 2.3 times that of the bacterial community, suggesting that the selenium sulfide shampoo primarily targets the fungal microbiota. Previous investigations have reported that individuals with dandruff or seborrheic dermatitis tend to exhibit higher bacterial diversity but reduced fungal diversity compared with healthy individuals (28, 31, 32). Therefore, these combined macro-ecological shifts observed in the present study indicate a treatment-associated restructuring of the scalp microbiome toward a healthier state.

Safety findings from the present study further support the clinical utility of the low-concentration formulation. No treatment-related adverse events, including edema, dryness, or stinging, were reported throughout the study period. Moreover, symptoms associated with scalp irritation, such as pruritus and papules, demonstrated significant improvement during treatment. Consistent with these findings, more than 90% of participants reported that the 0.5% selenium sulfide shampoo was mild and non-irritating. These results contrast with the adverse reactions frequently associated with higher-concentration selenium sulfide formulations, which have been reported to cause scalp dryness, tightness, burning sensations, stinging, an increased risk of contact dermatitis, and undesirable cosmetic effects such as excessive hair dryness or color fading in dyed hair (13, 14). Taken together, the present findings suggest that the 0.5% selenium sulfide formulation achieves a favorable balance between efficacy and tolerability. By maintaining therapeutic effectiveness while minimizing irritation and cosmetic concerns, this lower-concentration formulation may represent a gentler and more suitable treatment option for adolescents with dandruff or mild seborrheic dermatitis, potentially improving treatment adherence and supporting long-term symptom control.

To contextualize our findings, the overall design and objectives of the present trial closely resemble those of a recent pilot study by Wang et al. (33), which evaluated a 6% glycyrrhetinic acid complex shampoo for scalp seborrheic dermatitis. Consistent with their clinical outcomes, our 0.5% selenium sulfide intervention yielded significant reductions in scaling, erythema, and pruritus, alongside an excellent safety profile. Furthermore, both trials underscore the therapeutic importance of modulating the scalp microenvironment. While Wang et al. reported beneficial clinical and microbial shifts using a botanical-derived complex (33), our data demonstrate that a low-concentration selenium sulfide formulation achieves comparable microecological rebalancing by driving dysbiotic bacterial and fungal ratios toward a healthier profile. These parallel outcomes highlight that restoring scalp microbial homeostasis, supported by a well-tolerated formulation, is a central mechanism in the effective management of dandruff and seborrheic conditions.

Several limitations of the present study should be acknowledged. First, the single-arm study design and absence of a control group limit the ability to directly compare the observed effects with those of alternative treatments or placebo, thereby restricting the strength of causal inference. Additionally, the relatively small sample size may limit the generalizability of the findings to broader populations. And the attrition of 11 out of 42 initially enrolled participants may introduce selection bias and may limit the generalizability of the findings to broader populations. Thirdly, the short 4-week follow-up period restricts the evaluation of long-term therapeutic efficacy, disease recurrence, and the extended safety profile of the formulation. Finally, V3–V4 16S sequencing cannot fully resolve skin bacterial species like Staphylococcus, so our genus-level findings need high-resolution validation in further cohort studies. Consequently, future randomized controlled trials incorporating larger cohorts and prolonged follow-up periods are warranted to validate the feasibility of long-term management.

## 5 Conclusions

In conclusion, the present study demonstrates that a 0.5% selenium sulfide shampoo can significantly improve key clinical manifestations of dandruff in adolescents, including scaling, erythema, pruritus and scalp oiliness, and is associated with beneficial modulation of scalp microbial community structure toward a healthier profile, without inducing treatment-related adverse events. Taken together, these findings suggest that this low-concentration formulation represents an effective and well-tolerated topical therapeutic option for the management of adolescent dandruff.

## Data Availability

The datasets presented in this study can be found in online repositories. The repository name and accession number are: NCBI Sequence Read Archive (SRA), BioProject Accession Number [PRJNA1493314 and PRJNA1493320].

## References

[1] Sheth U Dande P. Pityriasis capitis: causes, pathophysiology, current modalities, and future approach. J Cosmet Dermatol. (2021) 20:35–47. doi: 10.1111/jocd.13488 32416039

[2] Chan CS Smith T He Z Garter C. The sequelae and moderators of influence of dandruff on mental health among mainland Chinese adults. Clin Cosmet Investig Dermatol. (2024) 17:1333–46. doi: 10.2147/CCID.S459498 38881702 PMC11179641

[3] Borda LJ Wikramanayake TC. Seborrheic dermatitis and dandruff: a comprehensive review. J Clin Investig Dermatol. (2015) 3:1000019. doi: 10.13188/2373-1044.1000019 27148560 PMC4852869

[4] Misery L Rahhali N Duhamel A Taieb C. Epidemiology of dandruff, scalp pruritus and associated symptoms. Acta Derm Venereol. (2013) 93:80–1. doi: 10.2340/00015555-1315 22277979

[5] Nawaf A Junaidu S Ibrahim S Babangida I Liadi S. Prevalence of dandruff among the pupils and staff of some selected public schools in Katsina State. UMYU Scientifica. (2023) 2:121–7. doi: 10.56919/usci.2323.018

[6] Polaskey MT Chang CH Daftary K Fakhraie S Miller CH Chovatiya R. The global prevalence of seborrheic dermatitis: a systematic review and meta-analysis. JAMA Dermatol. (2024) 160:846–55. doi: 10.1001/jamadermatol.2024.1987 38958996 PMC11223058

[7] Breunig Jde A de Almeida HL Jr Duquia RP Souza PR Staub HL. Scalp seborrheic dermatitis: prevalence and associated factors in male adolescents. Int J Dermatol. (2012) 51:46–9. doi: 10.1111/j.1365-4632.2011.04964.x 22182377

[8] Zhang H Liao W Chao W Chen Q Zeng H Wu C . Risk factors for sebaceous gland diseases and their relationship to gastrointestinal dysfunction in Han adolescents. J Dermatol. (2008) 35:555–61. doi: 10.1111/j.1346-8138.2008.00523.x 18837699

[9] Kelly KA Balogh EA Kaplan SG Feldman SR. Skin disease in children: effects on quality of life, stigmatization, bullying, and suicide risk in pediatric acne, atopic dermatitis, and psoriasis patients. Children. (2021) 8:1057. doi: 10.3390/children8111057 34828770 PMC8619705

[10] Golics CJ Basra MKA Finlay AY Salek MS. Adolescents with skin disease have specific quality of life issues. Dermatology. (2009) 218:357–66. doi: 10.1159/000205524 19246884

[11] Adalsteinsson JA Kaushik S Muzumdar S Guttman-Yassky E Ungar J. An update on the microbiology, immunology and genetics of seborrheic dermatitis. Exp Dermatol. (2020) 29:481–9. doi: 10.1111/exd.14091 32125725

[12] Expert Committee for Hair Quality Control of National Health Commission. Consensus on the diagnosis and treatment of seborrheic dermatitis: integration of traditional Chinese and western medicine (2024 Edition) [in Chinese]. Chin J Dermatol Venereol. (2025) 39:1–9.

[13] Danby FW Maddin WS Margesson LJ. Rosenthal D. A randomized, double-blind, placebo-controlled trial of ketoconazole 2% shampoo versus selenium sulfide 25% shampoo in the treatment of moderate to severe dandruff. J Am Acad Dermatol. (1993) 29:1008–12. doi: 10.1016/0190-9622(93)70282-X 8245236

[14] Gilbertson K Jarrett R Bayliss SJ Berk DR. Scalp discoloration from selenium sulfide shampoo: a case series and review of the literature. Pediatr Dermatol. (2012) 29:84–8. doi: 10.1111/j.1525-1470.2011.01410.x 21453309

[15] Wei SY Zhang HY Yin YT Ma LJ Li L Dong YM . Factor analysis approach unveils the influencing factors of dandruff in the normal teenage population. Dermatol Ther. (2020) 33:e13690. doi: 10.1111/dth.13690 32468693

[16] Bacon RA Mizoguchi H Schwartz JR. Assessing therapeutic effectiveness of scalp treatments for dandruff and seborrheic dermatitis, part 1: a reliable and relevant method based on the adherent scalp flaking score (ASFS). J Dermatolog Treat. (2014) 25:232–6. doi: 10.3109/09546634.2012.687089 22515728

[17] D'Souza P Rathi SK. Shampoo and conditioners: what a dermatologist should know? Indian J Dermatol. (2015) 60:248–54. doi: 10.4103/0019-5154.156355 26120149 PMC4458934

[18] Godse G Godse K.. Safety, Efficacy and attributes of 2.5% selenium sulfide shampoo in the treatment of dandruff: a single-center study. Cureus. (2024) 16:e57148. doi: 10.7759/cureus.57148 38681430 PMC11055963

[19] Turcu G Artenie C Nowicka D Arenbergerova M Lazaridou E Khobzei K . Selenium disulfide-based shampoo applied for 4 weeks significantly improves dandruff and seborrheic dermatitis. Eur J Dermatol. (2023) 33:19–23. doi: 10.1684/ejd.2023.4402 37098775

[20] Wang L Liu H Li N Wang Y Liu Q Zheng Y . Effectiveness and tolerance of medicated shampoo containing selenium sulfide and salicylic acid in patients with seborrheic dermatitis. J Dermatolog Treat. (2025) 36:2506676. doi: 10.1080/09546634.2025.2506676 40421970

[21] Reygagne PE Deloche-Bensmaine C Leclerc-Mercier S Faure J Kovylkina N Clavaud C . A selenium disulfide-based shampoo is beneficial for dandruff management and rebalancing the scalp microbiome of subjects of any hair type. Skin Appendage Disord. (2025):1–12. doi: 10.1159/000544742 40385516 PMC12084028

[22] Massiot P Clavaud C Thomas M Ott A Gueniche A Panhard S . Continuous clinical improvement of mild-to-moderate seborrheic dermatitis and rebalancing of the scalp microbiome using a selenium disulfide-based shampoo after an initial treatment with ketoconazole. J Cosmet Dermatol. (2022) 21:2215–25. doi: 10.1111/jocd.14362 34416081 PMC10234447

[23] Barbosa V Melo DF Vañó-Galván S Lutchmanen-Kolanthan V Sant'Anna B Leclerc-Mercier S . A comparative randomized clinical study assessing the efficacy of a 1% selenium disulfide-based shampoo versus 2% ketoconazole shampoo in subjects with moderate to severe scalp seborrheic dermatitis. Skin Appendage Disord. (2024) 10:497–504. doi: 10.1159/000539209 39659649 PMC11627539

[24] McGinley KJ Leyden JJ Marples RR Kligman AM. Quantitative microbiology of the scalp in non-dandruff, dandruff, and seborrheic dermatitis. J Invest Dermatol. (1975) 64:401–5. doi: 10.1111/1523-1747.ep12512335 237965

[25] Saxena R Mittal P Clavaud C Dhakan DB Hegde P Veeranagaiah MM . Comparison of healthy and dandruff scalp microbiome reveals the role of commensals in scalp health. Front Cell Infect Microbiol. (2018) 8:346. doi: 10.3389/fcimb.2018.00346 30338244 PMC6180232

[26] Clavaud C Jourdain R Bar-Hen A Tichit M Bouchier C Pouradier F . Dandruff is associated with disequilibrium in the proportion of the major bacterial and fungal populations colonizing the scalp. PLoS ONE. (2013) 8:e58203. doi: 10.1371/journal.pone.0058203 23483996 PMC3590157

[27] Meisel JS Hannigan GD Tyldsley AS SanMiguel AJ Hodkinson BP Zheng Q . Skin microbiome surveys are strongly influenced by experimental design. J Invest Dermatol. (2016) 136:947–56. doi: 10.1016/j.jid.2016.01.016 26829039 PMC4842136

[28] Park T Kim HJ Myeong NR Lee HG Kwack I Lee J . Collapse of human scalp microbiome network in dandruff and seborrhoeic dermatitis. Exp Dermatol. (2017) 26:835–8. doi: 10.1111/exd.13293 28094891

[29] Liang B Pu M Xu YN Li Y Sun JN Zhu Y . Dandruff scalp microbiome exhibits flake severity and sex-related differences. Br J Dermatol. (2025) 193:ii32–9. doi: 10.1093/bjd/ljaf099 41118316

[30] Tao R Li R Wang R. Skin microbiome alterations in seborrheic dermatitis and dandruff: a systematic review. Exp Dermatol. (2021) 30:1546–53. doi: 10.1111/exd.14450 34415635

[31] Soares RC Camargo-Penna PH de Moraes VC De Vecchi R Clavaud C Breton L . Dysbiotic bacterial and fungal communities not restricted to clinically affected skin sites in dandruff. Front Cell Infect Microbiol. (2016) 6:157. doi: 10.3389/fcimb.2016.00157 27909689 PMC5112237

[32] Lin Q Panchamukhi A Li P Shan W Zhou H Hou L . *Malassezia* and *Staphylococcus* dominate scalp microbiome for seborrheic dermatitis. Bioprocess Biosyst Eng. (2021) 44:965–75. doi: 10.1007/s00449-020-02333-5 32219537

[33] Wang HC Wang CS Hsieh SC Hung YT Chen HH. Evaluation of a new-formula shampoo containing 6% glycyrrhetinic acid complex for scalp seborrheic dermatitis: a pilot study. J Cosmet Dermatol. (2022) 21:3423–30. doi: 10.1111/jocd.14623 34792270 PMC9542316

