# Supplementary material for: Clinical effects of a 0.5% selenium sulfide shampoo on dandruff and scalp microbiota in adolescents: a prospective single-arm clinical study
Source: Front Med (Lausanne). 2026 Jul 23;13:1866777. doi: 10.3389/fmed.2026.1866777 (PMC13441831; doi:10.3389/fmed.2026.1866777)
Supplement: Supplementary file 1 [file Table_1.docx]

***Supplementary materials***

Table S1. Changes in clinical efficacy parameters during the 4-week treatment period

| Parameter | Week 0 | Week 1 | Week 2 | Week 4 |
| --- | --- | --- | --- | --- |
| Dandruff score | 566, 16 (12, 40) | 396, 11 (7, 30)*** | 237, 6 (0, 38)*** | 194, 6 (1, 23)*** |
| Scalp oiliness score | 153, 5 (3, 7) | 144, 5 (3, 7) | 145, 4 (3, 6) | 127, 4 (3, 6)*** |
| Hair lift score | 107, 4 (2, 5) | 115, 4 (2, 5) | 112, 4 (2, 5) | 122, 4 (2, 6)** |
| Erythema score | 387, 12(6, 26) | 275, 10 (0, 18)** | 123, 3 (0, 15)*** | 77, 2 (0, 11)*** |
| Scalp pruritus score | 109, 3 (0, 7) | 60, 2 (0, 6)*** | 48, 1 (0, 6)*** | 37, 1 (0, 4)*** |

Data are presented as total score, median (minimum, maximum).

* P < 0.05; ** P < 0.01; *** P < 0.001, compared with baseline (week 0).

Table S2. Participant-reported perceived benefits and user experience during product use

| Parameter | Immediate (2 h) | Week 1 | Week 2 | Week 4 |
| --- | --- | --- | --- | --- |
| Perceived significant reduction in dandruff | 26 (83.87) | 27 (87.10) | 28 (90.32) | 27 (87.10) |
| Perceived relief from scalp itching | 29 (93.55) | 28 (90.32) | 28 (90.32) | 29 (93.55) |
| Perceived non-oily scalp/hair | 25 (80.65) | 25 (80.65) | 24 (77.42) | 26 (83.87) |
| Perceived clean and refreshed scalp | 22 (70.97) | 24 (77.42) | 29 (93.55) | 28 (90.32) |
| Perceived relief from scalp erythema | 22 (70.97) | 24 (77.42) | 24 (77.42) | 28 (90.32) |
| Repaired scalp barrier issues | - | 22 (70.97) | 25 (80.65) | 24 (77.42) |
| Perceived overall improvement in scalp health | - | 27 (87.10) | 26 (83.87) | 28 (90.32) |
| Perceived scalp non-dryness | 21 (67.74) | 20 (64.52) | 23 (74.19) | 24 (77.42) |
| Perceived scalp was more hydrated | 15 (48.39) | 17 (54.84) | 20 (64.52) | 24 (77.42) |
| Perceived relief from scalp odor | 27 (87.10) | 25 (80.65) | 26 (83.87) | 26 (83.87) |
| Product was mild and non-irritating | 30 (96.77) | 30 (96.77) | 29 (93.55) | 29 (93.55) |
| Product rinsed off easily | 30 (96.77) | 29 (93.55) | 30 (96.77) | 30 (96.77) |
| Liked the fragrance of the product | 23 (74.19) | 24 (77.42) | 26 (83.87) | 23 (74.19) |
| Product use alleviated scalp discomfort | 27 (87.10) | 27 (87.10) | 28 (90.32) | 30 (96.77) |
| Product had soothing efficacy | 26 (83.87) | 29 (93.55) | 27 (87.10) | 29 (93.55) |
| Hair felt voluminous/lifted after use | 23 (74.19) | 25 (80.65) | 27 (87.10) | 26 (83.87) |
| Long-lasting oil control effect (≥24 h but <48 h) | - | 22 (70.97) | 26 (83.87) | 27 (87.10) |
| Long-lasting oil control effect (≥48 h) | - | 14 (45.16) | 18 (58.06) | 18 (58.06) |
| Long-lasting oil control effect (≥72 h) | - | - | - | 19 (61.29) |
| Long-lasting itch relief effect (≥72 h) | - | - | - | 23 (74.19) |
| Subjective sensation of improved scalp condition | 25 (80.65) | 26 (83.87) | 25 (80.65) | 29 (93.55) |
| Product produced rich lather | 28 (90.32) | 29 (93.55) | 27 (87.10) | 30 (96.77) |
| Overall satisfaction with product | 31 (100) | 29 (93.55) | 29 (93.55) | 28 (90.32) |
| Product deeply cleansed the scalp | 22 (70.97) | 22 (70.97) | 28 (90.32) | 26 (83.87) |
| Product provided rapid dandruff relief | 21 (67.74) | 23 (74.19) | 25 (80.65) | 28 (90.32) |
| Improved oily and limp hair | 22 (70.97) | 25 (80.65) | 24 (77.42) | 27 (87.10) |
| Scalp felt a cooling sensation | 23 (74.19) | 25 (80.65) | 28 (90.32) | 26 (83.87) |
| Hair felt smooth | 24 (77.42) | 25 (80.65) | 23 (74.19) | 26 (83.87) |
| Hair appeared shiny | 20 (64.52) | 21 (67.74) | 25 (80.65) | 25 (80.65) |

Data are presented as n (%). n represents the number of participants who responded “agree” or “strongly agree” to each questionnaire item. Percentages were calculated as the number of participants selecting “agree” or “strongly agree” divided by the total number of participants.

Table S3. Changes in instrument-based scalp measurements during the 4-week treatment period

| Parameter | Week 0 | Week 1 | Week 2 | Week 4 |
| --- | --- | --- | --- | --- |
| Sebum spot percentage (%) | 19.87 ± 12.41 | 22.60 ± 12.46 | 17.99 ± 11.30 | 20.86 ± 14.04 |
| Average sebum spot size (pixels) | 953.65 ± 892.50 | 978.06 ± 984.69 | 692.33 ± 456.19* | 833.13 ± 902.39 |
| Transepidermal water loss (g/m²·h) | 35.66 ± 8.18 | 32.10 ± 5.60* | 32.39 ± 6.75* | 31.12 ± 4.36** |
| Stratum corneum hydration (a.u.) | 30.49 ± 10.28 | 32.33 ± 10.54 | 33.77 ± 10.14* | 35.01 ± 9.64*** |

Data are presented as mean ± standard deviation.

* P < 0.05; ** P < 0.01; *** P < 0.001, compared with baseline (week 0).

Table S4. Changes in the relative abundance of scalp bacterial and fungal taxa after 4 weeks of treatment

| Microbiota | Week 0 | Week 4 | P value |
| --- | --- | --- | --- |
| *Cutibacterium* | 0.40 ± 0.23 | 0.63 ± 0.31 | <0.001 |
| *Staphylococcus* | 0.41 ± 0.22 | 0.20 ± 0.21 | <0.001 |
| *Malassezia* | 0.92±0.17 | 0.77±0.31 | <0.01 |
| *Malassezia restricta* | 0.69 ± 0.31 | 0.32 ± 0.37 | <0.001 |
| *Malassezia globosa* | 0.003 ± 0.015 | 0.006 ± 0.015 | <0.05 |

Data are presented as mean ± standard deviation.

Table S5. Alpha diversity indices of the scalp microbiome at baseline and after 4 weeks of treatment

| Microbiota | Time point | Shannon index | Simpson index | Pielou evenness index |
| --- | --- | --- | --- | --- |
| Bacteria | Week 0 | 2.08 ± 1.17 | 0.60 ± 0.17 | 0.38 ± 0.13 |
|  | Week 4 | 1.81 ± 1.45** | 0.46 ± 0.25*** | 0.31 ± 0.18** |
| Fungi | Week 0 | 0.85 ± 0.75 | 0.35 ± 0.26 | 0.23 ± 0.17 |
|  | Week 4 | 1.38 ± 1.05** | 0.47 ± 0.28* | 0.33 ± 0.22* |

Data are presented as mean ± standard deviation.

* P < 0.05; ** P < 0.01; *** P < 0.001, compared with baseline (week 0).

Table S6. Complete ingredient list of the 0.5% selenium sulfide shampoo.

Ingredients

Water, Sodium C14-16 Olefin Sulfonate, Sodium Lauroyl Sarcosinate, Lauryl Hydroxysultaine, Sodium Lauroamphoacetate, Parfum, Salicylic Acid, Cocamidopropyl Betaine, Carbomer, Sodium Benzoate, Phenoxyethanol, Menthol, Selenium Disulphide, Glycerin, Sodium Chloride, C12-13 Alkyl Lactate, Sodium Hydroxide, Butylene Glycol, Polyquaternium-10, Citric Acid, Colloidal Sulfur, Portulaca Oleracea Extract, 10-Hydroxydecanoic Acid, PPG-13-Decyltetradeceth-24, Panthenol, Phytosterols, Hexadimethrine Chloride, Zinc PCA, Starch Hydroxypropyltrimonium Chloride, Dichlorobenzyl Alcohol, Disodium Phosphate, Methyl Cyclodextrin, Acacia Senegal Gum, Maltodextrin

Table S7. Detailed scoring criteria for investigator-assessed clinical efficacy parameters.

| Parameter | Score | Clinical Description / Anchors |
| --- | --- | --- |
| Dandruff Severity (ASFS) | 0 | No dandruff. |
|  | 1-2 | Small, powdery, grayish-white rough scales. |
|  | 3-4 | Small to medium-sized flakes. |
|  | 5-6 | Large, thin flakes loosely attached to the scalp. |
|  | 7-8 | Large adherent flakes. |
|  | 9-10 | Thick white to yellow scales tightly adhered to the scalp. |
| Scalp Oiliness | 0 | Dry scalp without sebum or shine. |
|  | 1-2 | Slight moistness in partial areas with negligible oiliness. |
|  | 3-4 | Mild oiliness with faint shine and no hair clumping. |
|  | 5-6 | Moderate oiliness with obvious shine; hair roots attach to the scalp and slight clumping occurs. |
|  | 7-8 | Severe oiliness, prominent sebum accumulation and extensive hair matting. |
|  | 9 | Extreme greasiness with heavy sebum coverage; hair mats tightly against the scalp. |
| Scalp Erythema | 0 | No erythema; capillaries are normal. |
|  | 1-2 | Minimal and slight focal erythema. |
|  | 3-4 | Mild to mild-moderate erythema with partial distribution. |
|  | 5-6 | Moderate to moderate-severe erythema with capillary congestion. |
|  | 7-8 | Severe to very severe diffuse erythema and inflammation. |
|  | 9 | Extreme diffuse erythema with severe congestion and irritation. |
| Hair Lift | 0 | Completely flat against the scalp. All hair lies tightly on the scalp with no volume at all, and there is no inter-fiber space. |
|  | 1-2 | Severely flattened. Most or all of the hair mass adheres to the scalp and collapses obviously, and there is barely visible or no inter-fiber space. |
|  | 3-4 | Slightly flattened to flattened. The hair lies close to the scalp with weak or no obvious volume, and there is tiny or narrow inter-fiber gaps. |
|  | 5-6 | Moderately to slightly voluminous. Mild to steady natural fluffiness can be observed, and there is obvious or small gaps between fibers. |
|  | 7-8 | Very voluminous. The hair looks very airy overall, with only minor local flattening at most, and there is still ample inter-fiber space. |
|  | 9 | Extremely voluminous. Hair stands fully upright against the scalp with optimal lift, and there is ample inter-fiber space. |

Table S8. Additional ratio analyses of scalp microbial markers at baseline (BL) and week 4 (W4).

| Index | BL | W4 | P Value |
| --- | --- | --- | --- |
| *Cutibacterium / Staphylococcus*  (genus level) | 2.41±4.61 | 38.12±116.03 | <0.001 |
| log *(Malassezia restricta+0.001) /( Malassezia globosa+0.001）*  (species level) | 8.68±1.82 | 5.76±2.99 | <0.001 |

Notes: Data are presented as mean ± SD. The species-level ratio was calculated as log2[(*Malassezia restricta+0.001*)/(*Malassezia globosa+0.001*)]. Statistical comparisons were performed using the Wilcoxon rank-sum test..

Table S9. Pseudocount sensitivity analysis for the paired log-ratio difference.

|  | **Median (Q1-Q3)** | | **Paired W4 vs BL** | | |  |
| --- | --- | --- | --- | --- | --- | --- |
| **Pseudocount** | **BL median (Q1-Q3)** | **W4 median (Q1-Q3)** | **Median paired diff.** | **HL estimate (95% CI)** | **P value** | **Direction** |
| 0.001 | 9.466 (8.615-9.808) | 5.517 (3.661-7.758) | -2.326 | -2.681 (-3.704 to -1.960) | <0.001 | W4 lower than BL |
| 0.010 | 6.330 (5.558-6.585) | 3.507 (1.839-5.307) | -2.071 | -2.107 (-2.856 to -1.404) | <0.001 | W4 lower than BL |
| 0.050 | 4.078 (3.448-4.327) | 1.866 (0.644-3.608) | -1.651 | -1.585 (-2.125 to -1.012) | <0.001 | W4 lower than BL |
| 0.100 | 3.161 (2.586-3.397) | 1.216 (0.358-2.812) | -1.448 | -1.293 (-1.725 to -0.827) | <0.001 | W4 lower than BL |
| 1.000 | 0.844 (0.591-0.966) | 0.179 (0.040-0.779) | -0.421 | -0.393 (-0.518 to -0.239) | <0.001 | W4 lower than BL |
| *Note: The log2 ratio was calculated as log2((M. restricta + pseudocount)/(M. globosa + pseudocount)). Values are presented as median (Q1-Q3). Diff. = median paired difference (W4 - BL); HL = Hodges-Lehmann estimate from paired Wilcoxon signed-rank test. P values correspond to the paired W4 vs BL comparison.* | | | | | | |

Because *M. globosa* contained multiple zero values, the relative abundance of *M. restricta* to *M. globosa* was summarized using a pseudocount-adjusted log2 ratio, calculated as log2((*M. restricta* + pseudocount)/(*M. globosa* + pseudocount)). With the primary pseudocount of 0.001, the median log2 ratio decreased from 9.466 at BL to 5.517 at W4, with a median paired difference of -2.326 (W4 - BL; Wilcoxon signed-rank test, P < 0.001). This decrease was consistent across all tested pseudocounts.

Figure S1. Representative images of dandruff lesions during treatment. Representative scalp images showing changes in dandruff severity following use of the test product, captured using the Skin-Visioscan® VC20Plus imaging system.

| **Visit** | **Participant No. 8** | **Participant No.** |
| --- | --- | --- |
| Week 0 |  |  |
| Week 1 |  |  |
| Week 2 |  |  |
| Week 4 |  |  |

Figure S2. Representative dermoscopic images of scalp erythema during treatment. Representative dermoscopic images demonstrating changes in scalp erythema following use of the test product, obtained using the I-scope dermatoscope.

| **Visit** | **Participant No. 1** | **Participant No. 22** |
| --- | --- | --- |
| Week 0 |  |  |
| Week 1 |  |  |
| Week 2 |  |  |
| Week 4 |  |  |

Figure S3. Additional analyses of scalp microbial ratios between baseline (BL) and week 4 (W4). (A) Genus-level *Cutibacterium/Staphylococcus* ratio at baseline and week 4.. (B) Species-level *Malassezia restricta/Malassezia globosa* ratio, calculated as log2[(M. restricta+0.001)/(M. globosa+0.001)], at the species level. Data are presented as mean ± standard error. Statistical analyses were performed using the Wilcoxon rank-sum test. Differences with P < 0.001 were considered statistically significant.

Figure S4. Sensitivity of the median paired log-ratio difference to pseudocount selection. Sensitivity analysis of the median paired log-ratio difference across pseudocount values. For each pseudocount, the log-ratio was calculated as log2((M. restricta + pseudocount)/(M. globosa + pseudocount)), and the paired difference was defined as W4 - BL. The median paired difference remained below zero across all tested pseudocounts, indicating a consistently lower M. restricta-to-M. globosa log-ratio at W4 than at BL.
